# HyProCure for Pediatric Flexible Flatfoot: What Affects the Outcome

**DOI:** 10.3389/fped.2022.857458

**Published:** 2022-04-14

**Authors:** Cheng Chen, JianTao Jiang, ShaoLing Fu, Cheng Wang, Yan Su, GuoHua Mei, JianFeng Xue, Jian Zou, XueQian Li, ZhongMin Shi

**Affiliations:** Foot & Ankle Section, Department of Orthopaedics, Shanghai Jiao Tong University Affiliated Sixth Peoples Hospital, Shanghai, China

**Keywords:** pediatric flexible flatfoot, subtalar arthroereisis, HyProCure, risk factor, surgery

## Abstract

**Background:**

The high success rate, minimal invasion, and safety of subtalar arthroereisis (SA) have made it a primary mode of surgical management for pediatric flexible flatfoot. The HyProCure procedure is a new surgery for SA, However, very few available studies reported the therapeutic effects of the HyProCure procedure, especially in pediatric flexible flatfoot. The main aim of the present study was to investigate the clinical and radiological outcomes of the HyProCure procedure for pediatric flexible flatfoot and analyze the risk factors for therapeutic outcomes and sinus tarsi pain.

**Methods:**

In this retrospective cohort study, 69 pediatric flexible flatfoot patients (107 feet) who underwent the HyProCure procedure were included between July 2015 and September 2020. All patients underwent the HyProCure procedure with or without gastrocnemius recession. The Maryland foot score (MFS), visual analog scale (VAS), radiographic data, and complications were assessed at a minimum 1-year follow-up and statistically analyzed.

**Results:**

The mean follow-up was 35.9 months (range, 13–73 months). At the last follow-up, VAS (0.64 ± 1.16) was significantly lower than the preoperative VAS (4.06 ± 1.43) (*p* < 0.001); MFS (90.39 ± 12.10) was significantly higher than the preoperative MFS (71.36 ± 10.25) (*p* < 0.001). The AP talar-second metatarsal angle (T2MT angle) significantly decreased from 17.0 ± 5.4° preoperatively to 11.4 ± 5.2° at the last follow-up (*p* < 0.001). The lateral talar-first metatarsal angle (Meary's angle) significantly decreased from 13.8 ± 6.4° preoperatively to 6.3 ± 5.0° at the last follow-up (*p* < 0.001). The calcaneal declination angle (Pitch angle) significantly increased from 13.5 ± 4.9° preoperatively to 14.8 ± 4.4° at the last follow-up (*p* < 0.001). Logistic regression analysis indicated that patients with a longer distance from the tail end of the implant exceeding the longitudinal talar bisection line had 275.8% greater odds of MFS < 90. Yet, no risk factors were found in connection with sinus tarsi pain.

**Conclusions:**

The HyProCure procedure for pediatric flexible flatfoot achieved satisfactory curative effects with a low complication rate; implant depth was associated with unsatisfactory postoperative outcome.

## Introduction

Flatfoot is characterized by collapse of the medial longitudinal foot arch, with complex pathophysiological changes, including subluxation of subtalar joint, hindfoot valgus, midfoot abduction, and forefoot rotation. Meanwhile, contracture of gastrocnemius or Achilles tendon, often accompanied by flatfoot, further aggravates hindfoot valgus. Flatfoot in children and teenagers is rather common, defined as pediatric flatfoot. Importantly, a clear majority of pediatric flatfoot cases are flexible, characterized by foot arch collapsing during weight-bearing stress. Furthermore, Bresnahan and Graham described the deformity as recurrent and/or partial talotarsal joint dislocation (RTTJD) (1–3).

The incidence of pediatric flexible flatfoot was 22.6% on average, which decreased from 39.5% at 6 years to 11.8% at 12 years (4). Thereinto, a fair number of pediatric flexible flatfoot patients present with pain, fatigue, malalignment, and restriction of athletic activities. It is reported that the incidence of pathological flatfoot was 10.3% in children aged 7–14 years (5). Kothari et al. (6) observed that children with flatfoot tend to have more pain at the knee, hip, and back. Besides, the severity of flatfoot is likely related to incidence rate of knee pain and low back pain (7). However, no studies explain the mechanisms by which this occurs. Furthermore, a recent study found that flatfoot seems to have a potential association with spinal degeneration (8). Resolving foot symptoms, improving gait, restoring alignment, and reengaging in physical activities are the main goals of treatment. Conservative treatment, including observation, orthosis, and physical therapy, is the prior option that majority of clinicians would choose to relieve pain (9, 10). However, a failed trial of non-operative therapy indicates a surgical procedure. Surgical procedures include soft tissue procedures, bony procedures (osteotomies and arthrodesis), and arthroereisis (11–14).

Subtalar arthroereisis (SA), a minimally invasive procedure for flexible flatfoot, has been proven safe and to have satisfactory results with the advantage of rapid recovery and little influence on bone growth (11–21). Therefore, SA is gaining great popularity in the treatment of pediatric flexible flatfoot (9, 10, 22, 23). Thereinto, the HyProCure device (GraMedica, Macomb, MI, USA), a new extraosseous talotarsal stabilization (EOTTS) device (24), is receiving growing attention in its clinical application. Yet, there have been very few studies reporting clinical outcomes of the HyProCure procedure (1, 2, 25, 26) (Table 1). Sadly, only one of these studies makes mention of pediatric flexible flatfoot. Besides, because of adjunctive operative procedures, therapeutic results of the HyProCure procedure are hardly evaluated well.

**Table 1:** Clinical outcome of the HyProCure procedure.

**References**	**Patients (feet)**	**Follow-up, months**	**Age, years**	**MFS, points**	**AOFAS-AH, points**	**VAS, points**	**Sinus tarsi pain (rate)**	**Infection (rate)**	**Implant extrusion (rate)**	**Implant removal (rate)**
Merčun et al. (27)	87 (123)	30 (13–55)^*^	20.1 (6–75)^*^	/	/	5.5 ± 3.1 → 2.2 ± 2.5	11 (8.9%)	2 (1.6%)	6 (4.9%)	19 (15%)
Silva et al. (26)	31 (34)	24	46.9 ± 15.21^#^	/	50.3 ± 21.1 → 81.6 ± 21.6^#^	6.1 ± 3.0 → 1.4 ± 2.5^#^	7 (20.6%)	0	0	7 (20.6%)
Bresnahan et al. (2)	35 (46)	9 ± 5#	41 (8–72)^*^	69.53 ± 19.56 → 89.17 ± 14.41^#^	/	/	0	1 (2.86%)	0	2 (4.35%)
Graham et al. (1)	83 (117)	51 (38–65)^*^	58 (22–85)^*^	88 (31–100)^*^^†^	/	/	4 (3.4%)	1 (0.9%)	0	16 (13.7%)

*MFS, The Maryland Foot Score; AOFAS-AH, American Orthopedic Foot and Ankle Society Ankle-Hindfoot scale; VAS, visual analog scale*.

*MFS, AOFAS-AH, and VAS were presented as preoperative score → postoperative score*.

* *Variables were described as mean (range)*.

# *Variables were described as mean ± standard deviation*.

† *Only reported postoperative score*.

Recently, studies have focused on identifying factors associated with better outcomes for SA, inclusive of demographic data and specific disease parameters (28–30). To our knowledge, there is no available study on radiographic outcomes on the HyProCure procedure for pediatric flexible flatfoot and factors influencing clinical outcomes of the HyProCure procedure. Therefore, the purpose of our study was to explore the actual therapeutic results of the HyProCure procedure for pediatric flexible flatfoot and analyze risk factors associated with clinical outcome and sinus tarsi pain, which is the most common postoperative complication of SA.

## Methods

### Patients and Study Design

This retrospective cohort study included patients with pediatric flexible flatfoot receiving the HyProCure procedure at Shanghai Jiao Tong University Affiliated Sixth Peoples Hospital between July 2015 and September 2020. Patient medical records were reviewed *via* an electronic database. The inclusion criteria were as follows: (1) patients diagnosed as having symptomatic pediatric flexible flatfoot; (2) patients who underwent the HyProCure procedure for flatfoot; (3) patients aged between 10 and 18 years at the time of surgery; and (4) failure of 6 months' conservative treatment. The exclusion criteria were as follows: (1) rigid flatfoot patients; (2) neurological flatfoot patients; (3) patients treated with other procedures except for the HyProCure procedure and gastrocnemius recession; (4) patients with a history of previous flatfoot procedure; and (5) patients lost to follow-up, causing data loss of either clinical or radiological outcomes.

A total of 133 patients (196 feet) met the inclusion criteria, and 64 patients (89 feet) were discounted as per the exclusion criteria. Finally, 69 patients (107 feet) with complete data of clinical and radiological results were included in this study.

The study was approved by the Ethics Committee of Shanghai Jiao Tong University Affiliated Sixth Peoples Hospital and all patients provided informed consent preoperatively. The study is registered at Clinical Trials Registry (approval no. ChiCTR2100051519). The study was conducted in compliance with the Helsinki Declaration.

### Data Collection

**Figure 1:**
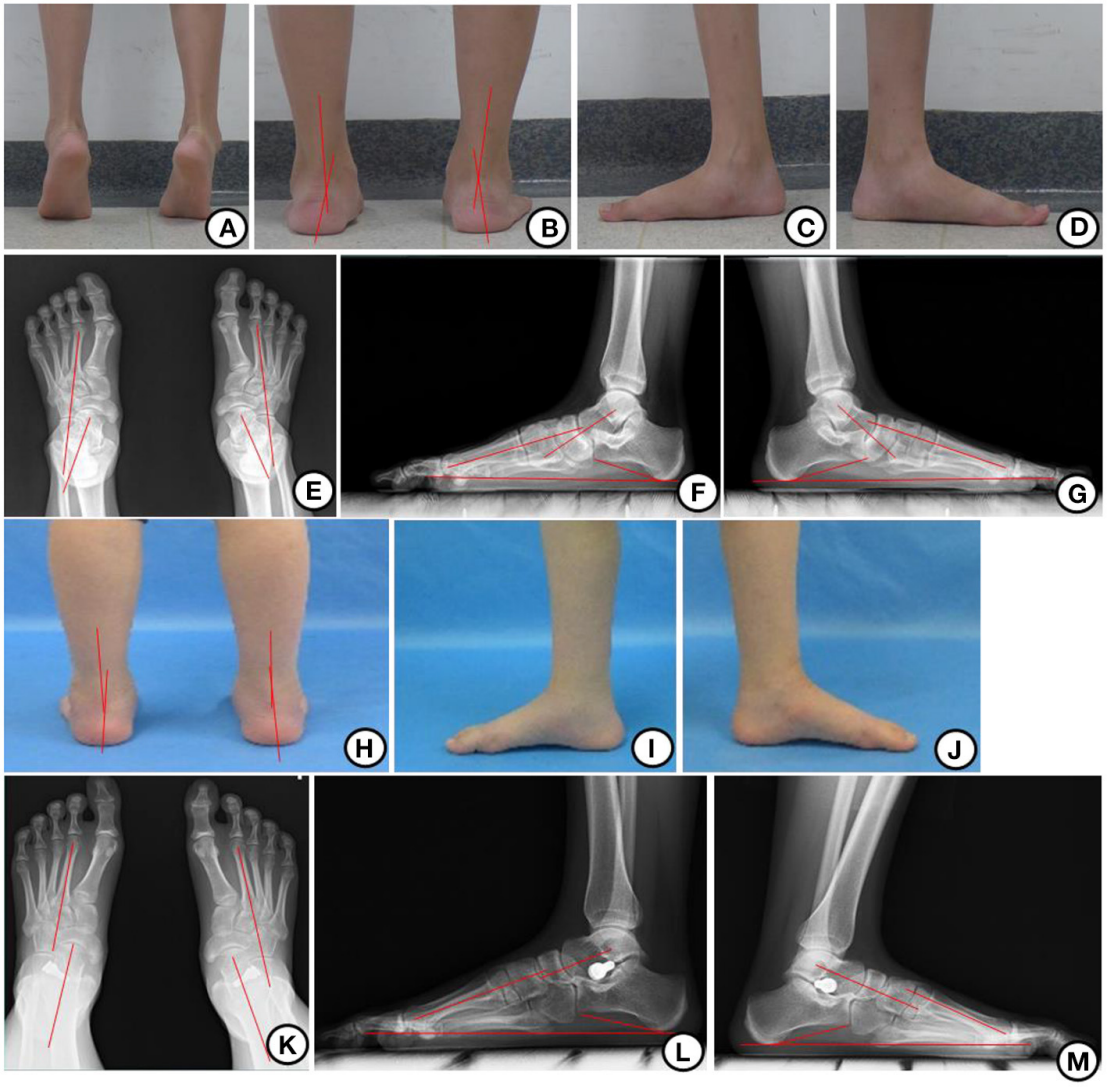
Appearance and x-ray before and after operation. **(A)** Hindfoot valgus could be corrected when calf raising before operation. **(B)** Hindfoot valgus before operation. **(C, D)** The medial appearance of left and right foot in one patient before the operation. **(E)** AP x-ray before operation showing T2MT angle. **(F, G)** Lateral x-ray before the operation showing Meary's and Pitch angle. **(H)** Hindfoot alignment was corrected 1 year after the operation. **(I, J)** The medial appearance of the left and right foot in one patient 1 year after the operation. **(K)** AP x-ray 1 year after the operation showing T2MT angle. **(L, M)** Lateral x-ray 1 year after the operation showing Meary's and Pitch angle.

For all participants, demographic characteristics and imaging data were recorded at baseline. Demographic data included gender, age, and body mass index (BMI). Other detailed data included follow-up time, disease side, gastrocnemius recession, as well as implant size and depth. Questionnaires regarding health status, including the Maryland Foot Score (MFS) and visual analog scale (VAS), were also obtained at baseline. MFS, generated on a scale of 0 to 100, is composed of 3 subscales, namely, pain, foot functional capability, and foot appearance, with higher scores indicating better results. The VAS for pain is a widely used tool for patients to measure pain intensity along a 10-cm line. Radiographic data included the AP talar-second metatarsal angle (T2MT angle), the lateral talar-first metatarsal angle (Meary's angle), and calcaneal declination angle (Pitch angle) (Figure 1). At the final postoperative follow-up, MFS, VAS, and radiographic data were evaluated. In addition, intraoperative and postoperative complications, including incision dehiscence, prolonged skin healing, infection, sinus tarsi pain, implant extrusion, and fracture, were recorded. All data were collected by 2 independent observers. If there were any discrepancies regarding the medical data, the senior physician would review the data and make the final decision.

### Surgical Technique

All patients underwent the HyProCure procedure with or without gastrocnemius recession. The patient was placed in a supine position on the operating table. For patients with a positive Silfverskiold test, a 2-cm posteromedial incision at the level of the musculotendinous junction was performed. Dissection was carried down to the posterior fascia taking care to identify and preserve the sural cutaneous nerve. The fascia was incised in line with the skin incision, and the gastrocnemius tendon was isolated from the underlying soleus. The distal end of the gastrocnemius aponeurosis was then cut sharply with a pair of scissors.

The HyProCure procedure approach used was an approximate 1.5-cm linear skin incision over the sinus tarsi at a distance of 1 cm from the distal aspect of the fibula. After blunt dissection creating a path, the soft tissues within tarsal sinus and canal were transected. A guidewire was gently inserted in the same direction as the tarsal sinus and canal. Trial sizing placed onto the guidewire was performed gradually to optimal size. Place the talotarsal joint through a full range of motion to decide on correction achieved *via* the corresponding trial sizer. The purpose was to restore the normal motion of 3° to 5° of hindfoot pronation. After determining the proper size, place the corresponding HyProCure device on the insertion driver and move it along the guidewire. Once the placement of the device examined by intraoperative fluoroscopy was done, the driver and guidewire were removed. Close the incision with suture and wrap a dry, sterile compression dressing around the foot and ankle. All patients received postoperative walking boots and crutches.

### Postoperative Rehabilitation

Postoperative rehabilitation was divided into 2 stages. Stage 1 lasted from the first day to 2 weeks postoperatively. Patients were allowed limited weight-bearing on the foot with the help of a postoperative shoe and crutch, as tolerated, and the ankle was placed in 90° dorsiflexion for the duration of non-weight-bearing. Patients were instructed to elevate the foot and perform active muscle contraction. They performed ankle plantar flexion/dorsal extension exercises, increasing the number by 30 times a day until reaching 150 times a day. Stage 2 began 2 weeks postoperatively. At 2 weeks postoperatively, patients were allowed to full weight-bearing walk and to increase activity as tolerated. Patients were encouraged to transfer to and use new and supportive shoes as soon as tolerated. Patients' rehabilitation exercises were followed up at 2 weeks, 4 weeks, 3 months, and 1 year postoperatively in the outpatient room or by telephone.

### Statistical Analysis

All continuous variables were described as mean ± standard deviation, while qualitative variables were described as proportions. Statistical analysis was performed using SPSS software (version 26.0; IBM, Armonk, NY, USA). Two-tailed *t*-tests were used to compare continuous data. Logistic regression analysis was used to analyze risk factors for MFS < 90 and sinus tarsi pain, respectively. Regression candidates included age, gender, BMI, gastrocnemius recession, implant size, and implant depth and were analyzed as covariates. *p* < 0.05 was considered statistically significant.

### Follow-Up and General Results

A total of 69 patients (107 feet) were included in this study. The mean follow-up was 35.9 months (range, 13–73 months). Of the 69 patients, 48 (69.6%) were male and 21 (30.4%) were female, with a mean age at the time of surgery of 11.4 years (range, 10–18 years). Mean BMI at the time of surgery was 20.1 kg/m^2^ (range, 12.7–32.7 kg/m^2^). Of the 107 feet, the right foot was involved in 50 (46.7%) of the feet and the left foot was involved in 57 (53.3%). The HyProCure procedure was performed alone in 46 feet (43.0%), while the HyProCure procedure with gastrocnemius recession was performed in 61 feet (57.0%). The most used implant size was 8 (35 feet, 32.7%), compared with 5 (18 feet, 16.8%), 6 (19 feet, 17.8%), 7 (28 feet, 26.2%), and 9 (7 feet, 6.5%). Implant depth is defined as whether the tail end of the HyProCure implant exceeded the longitudinal talar bisection line (24). The tail end of the HyProCure implant exceeded the longitudinal talar bisection line in 12 feet, while not exceeding in 95 feet.

### Clinical and Radiographic Outcomes

During the final follow-up, significant improvements of VAS and MFS were observed (Table 2). At the last follow-up, VAS (0.64 ± 1.16) was significantly lower than the preoperative VAS (4.06 ± 1.43) (*p* < 0.001); MFS (90.39 ± 12.10) was significantly higher than the preoperative MFS (71.36 ± 10.25) (*p* < 0.001). Moreover, MFS-Pain, MFS-Function, and MFS-Appearance were also significantly higher (*p* < 0.001). Meanwhile, T2MT angle significantly decreased from 17.0 ± 5.4° preoperatively to 11.4 ± 5.2° at the last follow-up (*p* < 0.001); Meary's angle significantly decreased from 13.8 ± 6.4° preoperatively to 6.3 ± 5.0° at the last follow-up (*p* < 0.001), while Pitch angle significantly increased from 13.5 ± 4.9° preoperatively to 14.8 ± 4.4° at the last follow-up (*p* < 0.001) (Table 3).

**Table 2:** Comparison of clinical outcome between preoperation and last follow-up (*n* = 107).

	**VAS**	**MFS**	**MFS-pain**	**MFS-function**	**MFS-appearance**
Preoperation	4.06 ± 1.43	71.36 ± 10.25	34.72 ± 6.06	34.76 ± 2.58	4.46 ± 2.28
Last follow-up	0.64 ± 1.16	90.39 ± 12.10	41.22 ± 5.36	40.79 ± 6.06	8.38 ± 2.17
*t*-value	−9.255	14.732	9.629	9.698	13.986
*P*-value	<0.001^*^	<0.001^*^	<0.001^*^	<0.001^*^	<0.001^*^

*MFS, The Maryland Foot Score; VAS, visual analog scale*.

*All continuous variables are described as mean ± standard deviation*.

* *p-value is less than 0.05, which means the difference is significant*.

**Table 3:** Comparison of radiographic outcome between preoperation and last follow-up (*n* = 107).

	**T2MT (**°**)**	**Meary's (**°**)**	**Pitch (**°**)**
Pre-operation	17.0 ± 5.4	13.8 ± 6.4	13.5 ± 4.9
Last follow-up	11.4 ± 5.2	6.3 ± 5.0	14.8 ± 4.4
*t*-value	10.190	13.353	−4.806
*P*-value	<0.001^*^	<0.001^*^	<0.001^*^

*T2MT, Talar-second metatarsal*.

*All continuous variables are described as mean ± standard deviation*.

* *p-value is less than 0.05, which means the difference is significant*.

### Complications

As for complications, no intraoperative complications occurred. 1 patient (1 foot) developed prolonged skin healing, and 1 patient (1 foot) suffered from implant extrusion. 10 patients (14 feet) suffered from tarsal sinus pain, among which 7 patients (10 feet) felt symptomatic relief after adopting conservative treatment; 3 patients (4 feet) had to remove implants with conservative treatment being ineffective.

### Logistic Regression

Age, gender, BMI, gastrocnemius recession, implant size, and implant depth were analyzed as covariates. Logistic regression analysis showed that implant depth was the only risk factor associated with MFS < 90 with an odds ratio of 3.758 (*p* = 0.041, 95% CI: 1.053–13.412). In other words, patients with a longer distance from the tail end of the implant exceeding the longitudinal talar bisection line had 275.8% greater odds of MFS < 90. Besides, none of the above 6 covariates was the risk factor associated with sinus tarsi pain (Table 4).

**Table 4:** Logistic regression analysis of MFS < 90 and sinus tarsi pain.

	**MFS** **<** **90**		**Sinus tarsi pain**
	**Univariate logistic regression**	**Multivariate logistic regression**	**Logistic regression^#^**
	***P*** **-value**	***P*** **-value**	***P*** **-value**
Age	0.098	0.176	0.724
BMI	0.753	0.784	0.179
Gastrocnemius recession	0.175	0.304	0.555
Gender	0.449	0.277	0.103
Implant depth^※^	0.041^*^ (OR = 3.758, 95% CI: 1.053–13.412)	0.041^*^ (OR = 3.758, 95% CI: 1.053–13.412)	0.605
Implant size			
5	0.808	0.941	0.454
6	0.708	0.814	0.256
7	0.902	0.999	0.665
8	0.550	0.728	0.723
9	0.289	0.439	0.209

*MFS, The Maryland Foot Score; OR, odds ratio*.

# *For both univariate logistic regression and multivariate logistic regression*.

※ *Implant depth is defined as whether the tail end of the HyProCure implant exceeded the longitudinal talar bisection line (19)*.

* *p-value is less than 0.05, which means the difference is significant*.

## Discussion

In this study, we found that clinical and radiological results of the HyProCure procedure for pediatric flexible flatfoot were satisfactory. Further analysis indicated that implant depth was a risk factor for unsatisfactory postoperative efficacy, while age, BMI, gender, gastrocnemius recession, and implant size had little relationship with the efficacy. However, none of the above 6 factors was associated with sinus tarsi pain.

Almost every infant is born with painless flexible flatfoot, progressively developing a medial longitudinal arch during the first decade of their lives. As age grows, flatfoot gradually resolves. A study on footprint shows that the maturation of the medial longitudinal arch lasts about 4 years from age 6 to age 10 (31). The prevalence of flatfoot is 54% by age 3, decreasing to 26% by age 6, and ultimately down to 4% by age 10 with minimal variation (11). By the age of 10, most pediatric flatfoot resolves while, as for others, it persists into adolescence and adulthood. Therefore, we hold a conservative attitude toward surgery for children younger than 10 years. We agree with De Pellegrin et al. (20) that the patient should be at least 10 years of age at the time of surgery so that the growth potential of the foot can be fully utilized and to allow for spontaneous resolution, thereby avoiding the possibility of over-treatment. Conservative treatment, the primary management option (9, 10), is also our first choice, but for those who experienced failure of 6 months' conservative treatment, we choose to turn to surgery. In consideration of the uncertain effectiveness of conservative treatment (32), it may be pointless to prolong the course of conservative treatment after failure of 6 months' conservative treatment. Nevertheless, we admit that controversy about the treatment of pediatric flexible flatfoot still exists, which needs further study to verify.

Although SA showed an overall decent mid-term result (29, 33, 34), a long-term effect remains unclear. So far, only one study has reported long-term follow-up results, which lack conviction. Mazzotti et al. (30) conducted a study including 34 patients (64 feet), of which average follow-up was 180 ± 32.9 months (120 to 240). Long-term result as a whole was encouraging, yet only 18 patients (52.9%) performed sports activities regularly. In addition, long-term complication of SA, although rare, is still poorly evaluated and lacks comprehensive exposition (35–38). Moreover, up to now, no study has looked into the long-term impact of SA on the subtalar joint; thus, whether SA might increase the risk of subtalar osteoarthritis is still unknown.

An impact blocking device, the calcaneo-stop screw, and a self-locking device are the mainstream of SA implants in current practice (39). Memeo et al. (40) compared exosinotarsal arthroereisis with calcaneo-stop screw and endosinotarsal arthroereisis with self-locking device in treating pediatric flexible flatfeet. They found no significant differences in radiographic or clinical parameters between the two groups in the short and medium term. A recent systematic review (41) showed that both impact blocking and self-locking devices were proven to be valid and effective for treating pediatric flexible flatfeet. Nonetheless, for those with obesity or those demanding high performance in sports, the calcaneo-stop procedure may be better and more recommended (41). The calcaneo-stop procedure, a cheap, simple, and effective technique, has shown satisfactory clinical and radiographic outcomes and low rates of complications (42). However, removal of the calcaneo-stop screw, 2 years postoperatively or after skeletal maturity, needs a second operation.

The HyProCure device is of the self-locking mechanism type according to Vogler classification and type II EOTTS according to Graham classification (24). Importantly, the shape of the HyProCure device closely mimics the space and orientation of tarsal sinus and canal; hence, it anatomically fits. Specifically, the mechanism of the HyProCure device is that the talus glides over the device rather than hitting up against the device as a bone blocking procedure. Besides, the HyProCure device, made of medical-grade titanium alloy, is designed to remain in the body without routine removal. Moreover, finite element analysis and biomechanical assessment of a cadaveric study reported that the HyProCure device exhibited a more obvious effect of deformity correction than type I EOTTS (43, 44).

Sadly, very few available studies reported the therapeutic effects of the HyProCure procedure (1, 2, 26, 27) (Table 1); several defects more or less limit persuasiveness. Silva et al. (26) reported 2-year clinical and radiological results of the HyProCure procedure for symptomatic Grade II Adult acquired flat foot deformity (AAFD). They found that T1MT angle improved from 14.0 ± 2.6° preoperatively to 1.3 ± 2.4° postoperatively and Pitch angle improved from 8.4 ± 3.5° preoperatively to 22.7 ± 8.4° postoperatively. Graham's study (1), inducing 83 adult patients (117 feet) with the HyProCure procedure, included neither preoperative statistics nor preoperative and postoperative comparisons. Furthermore, Graham et al. (20) conducted a radiographic evaluation on 70 patients (95 feet) in the above study. In their study, T2MT angle significantly decreased from 24.8 ± 1.0° preoperatively to 5.8 ± 0.9° postoperatively, while Pitch angle slightly increased from 21 ± 0.7° preoperatively to 21.8 ± 0.7° postoperatively. However, postoperative radiographs were taken at an average follow-up of 17 days from the surgery date, which was too short for patients to recover yet, hardly performing weight-bearing radiographs. Bresnahan et al. (2) performed a prospective study; nonetheless, its biggest limitation was the short follow-up (the mean follow-up was 37.75 ± 20.49 weeks). It should be noted that none of the above studies focused on pediatric flexible flatfoot. Recently, Merčun et al. (27) reported a high satisfaction rate of 84%. What is more, 40 pediatric-isolated cases significantly showed the best outcome, whereas adults with combined procedures reported the lowermost outcome. However, this study lacked baseline subjective evaluation and only measured *via* Likert scale and VAS instead of a specific scoring system for foot and ankle.

Therefore, we worked on evaluating the HyProCure procedure for pediatric flexible flatfoot, with an effort to eliminate the impact of adjunctive operative procedures. To eliminate heterogeneity, our study included pediatric flexible flatfoot patients aged 10 to 18 years who underwent the HyProCure procedure with or without gastrocnemius recession, as gastrocnemius recession had a negligible impact on pediatric flexible flatfoot (34). Our study showed the satisfactory clinical outcomes of the HyProCure procedure for pediatric flexible flatfoot. Besides, our study exhibited vast improvements in Meary's angle and Pitch angle after surgery, which were both of statistical and clinical significance. Although we found that Pitch angle improvement showed a statistical difference, the numerical increase was too small to reach clinical significance. Of note, we found that implant depth was a risk factor for unsatisfactory postoperative efficacy, while age, BMI, gender, gastrocnemius recession, and implant size had nothing to do with the outcome, although previous studies suggested that age was an important influencing factor of postoperative outcomes for pediatric flexible flatfoot (20, 29, 45) and obesity had a correlation with implant extrusion and worse results after SA (28). On the contrary, our study indicated that age and BMI had no connection with unsatisfactory curative effect.

Sinus tarsi pain is the most common postoperative complication of ST. We found that the incidence of sinus tarsi pain was 13.1%, However, most of the sinus tarsi pain cases were alleviated after conservative treatment. Yet, the incidence of implant removal was 3.7%, which was on par with that in previous reports on the HyProCure procedure (1, 2, 24). The anatomical design of the HyProCure device fits with the tarsal sinus and canal and is placed along with its natural orientation, theoretically allowing for uniform force distribution and better biomechanical functioning (24). This effect may explicate low postoperative implant removal rates of the HyProCure device. Wang et al. (34) reported that patients with a longer distance from the tail end of the implant to the lateral calcaneal wall had 38.8% greater odds of developing sinus tarsi pain *via* multivariate logistic regression analysis. Saxena et al. (46) found that implant size was a factor for implant removal in adults (except for implant dislocation, 82.6% of implant removal was due to sinus tarsi pain). However, we failed to reveal the risk factors for sinus tarsi pain.

### Limitations of the Study

Several limitations of this study should be discussed. First, recall bias is unavoidable because of the retrospective design. Second, the relatively small sample size cannot provide more reliable results, especially concerning the occurrence of complications. Third, there is a shortage of short-term follow-up, and thus, the long-term results, essential for teenagers, still need to be evaluated.

## Conclusion

In conclusion, our retrospective cohort study demonstrated the predominant outcome and low complication rates of HyProCure for pediatric flexible flatfoot; implant depth was associated with unsatisfactory postoperative outcome.

## Data Availability Statement

The raw data supporting the conclusions of this article will be made available by the corresponding authors, on reasonable request.

## Ethics Statement

The studies involving human participants were reviewed and approved by Ethics Committee of Shanghai Jiao Tong University Affiliated Sixth Peoples Hospital. Written informed consent to participate in this study was provided by the participants' legal guardian/next of kin. Written informed consent was obtained from the minor(s)' legal guardian/next of kin for the publication of any potentially identifiable images or data included in this article.

## Author Contributions

ZS and CC conceptualized the study. JJ, XL, SF, and CW reviewed the literature and designed the figure and table. CC, YS, GM, JX, and JZ collected patients' information. CC drafted the manuscript. ZS, XL, and JJ revised the manuscript. All authors have read and approved the final version.

## Funding

This study was sponsored by Bio-medical Engineering Program of ‘Shanghai Jiao Tong University Star’ Plan (YG2022ZD018); Key Research and Development Program of National Ministry of Science and Technology (2018YFC2001504); Shanghai Artificial Intelligence Innovation and Development Program (2020-RGZN-02006); and Biomedicine Supporting Program of Shanghai ‘Science and Technology Innovation Plan’ (19441902400).

## Conflict of Interest

The authors declare that the research was conducted in the absence of any commercial or financial relationships that could be construed as a potential conflict of interest.

## Publisher's Note

All claims expressed in this article are solely those of the authors and do not necessarily represent those of their affiliated organizations, or those of the publisher, the editors and the reviewers. Any product that may be evaluated in this article, or claim that may be made by its manufacturer, is not guaranteed or endorsed by the publisher.
